# Benefit delayed immunosenescence by regulating CD4 ^+^T cells: A promising therapeutic target for aging‐related diseases

**DOI:** 10.1111/acel.14317

**Published:** 2024-08-18

**Authors:** Tingting Xia, Ying Zhou, Jiayao An, Zhi Cui, Xinqin Zhong, Tianyi Cui, Bin Lv, Xin Zhao, Xiumei Gao

**Affiliations:** ^1^ Ministry of Education Key Laboratory of Pharmacology of Traditional Chinese Medical Formulae Tianjin University of Traditional Chinese Medicine Tianjin China; ^2^ State Key Laboratory of Component‐Based Chinese Medicine Tianjin University of Traditional Chinese Medicine Tianjin China; ^3^ State Key Laboratory of Chinese Medicine Modernization Tianjin University of Traditional Chinese Medicine Tianjin China

**Keywords:** aging‐related diseases, effector CD4^+^T cells, immunosenescence, memory CD4^+^T cells, Naïve CD4^+^T cells

## Abstract

CD4^+^T cells play a notable role in immune protection at different stages of life. During aging, the interaction between the body's internal and external environment and CD4^+^T cells results in a series of changes in the CD4^+^T cells pool making it involved in immunosenescence. Many studies have extensively examined the subsets and functionality of CD4^+^T cells within the immune system, highlighted their pivotal role in disease pathogenesis, progression, and therapeutic interventions. However, the underlying mechanism of CD4^+^T cells senescence and its intricate association with diseases remains to be elucidated and comprehensively understood. By summarizing the immunosenescent progress and network of CD4^+^T cell subsets, we reveal the crucial role of CD4^+^T cells in the occurrence and development of age‐related diseases. Furthermore, we provide new insights and theoretical foundations for diseases targeting CD4^+^T cell subsets aging as a treatment focus, offering novel approaches for therapy, especially in infections, cancers, autoimmune diseases, and other diseases in the elderly.

## Graphical abstract

CD4^+^T cells play a notable role in immune protection at different stages of life. During aging, the interaction between the body's internal and external environment and CD4^+^T cells, results in a series of changes in the CD4^+^T cells pool making it involved in immunosenescence. CD4^+^T cells subsets aging as a treatment focus, offering novel approaches for therapy, especially in infections, cancers, autoimmune diseases, and other diseases in the elderly.

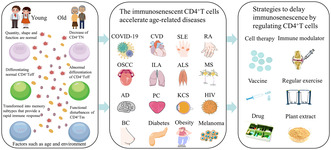

**Abbreviations**
AD: Alzheimer disease
AKT: protein kinase B
ALS: amyotrophic lateral sclerosis
BC: breast cancer
CD4^+^Teff: effector CD4^+^T cells
CD4^+^TEMRA: terminally differentiated CD4^+^T cells
CD4^+^Tm: Memory CD4^+^T cells
CD4^+^TN: naïve CD4^+^T cells
COVID‐19: corona virus disease 2019
CVD: cerebrovascular disease
DCs: dendritic cells
DNA‐PK: DNA dependent protein kinase
HIV: human immunodeficiency virus
HSCs: hematopoietic stem cells
ILA: Interstitial lung abnormality
KCS: malignant keratoconjunctivitis
LAG‐3: lymphocyte activation gene 3
MDSCs: myeloid‐derived suppressor cells
MS: multiple sclerosis
MSCs: mesenchymal stem cells
mTOR: rapamycin
mTORC1: rapamycin complex 1
mTORC2: mTOR complex 2
MΦ: monocyte macrophages
OSCC: oral squamous cells carcinoma
PC: prostate cancer
PI3K: phosphatidylinositol 3‐kinase
RA: rheumatoid arthritis
rIL‐37: recombinant interleukin‐37
rsIL‐7: recombinant interleukin‐7
SASP: senescence‐associated secretory phenotype
SLE: aystemic lupus erythematosus
sMAC: sestrins‐MAP kinase activated complex
STAT5: activator of transcription 5
STING: stimulator of interferon genes
TAB1: protein kinase binding protein 1
T‐bet: T‐box‐expressed‐in‐T‐cells
TCA: tricarboxylic acid cycle
TCR: T cells receptor
Tfh: T follicular helper
Th1: T helper type 1
Th17: T helper type 17
Th2: T helper type 2
ThPOK: T‐helper‐inducing POZ/Krueppel‐like factor
TREC: T cells receptor excision circle
Treg: regulatory T cell

## 1 INTRODUCTION

**FIGURE 1:**
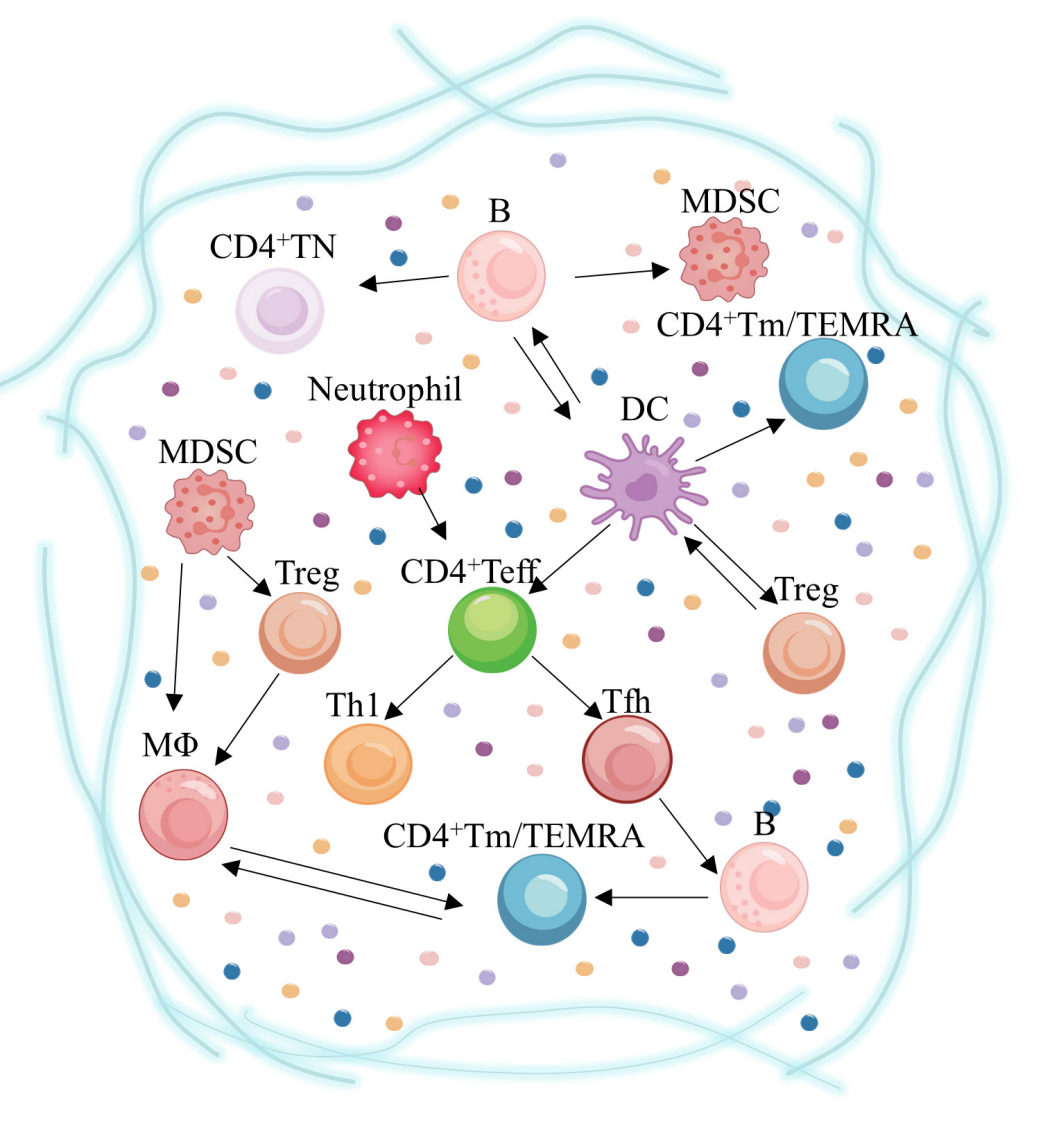
The key role of different subtypes of CD4^+^T cells in the immune microenvironment of age‐related diseases. Tregs are stimulated by DCs in age‐related diseases, downregulating the threshold for resistance to exogenous antigens and, in turn, maintaining the tolerant function of DCs. DCs promote CD4^+^Teff differentiation to Tfh by increasing cytokine secretion. Furthermore, IL‐6 secreted by DCs is dependent on enhanced responses of senescent CD4^+^TN. B cells influence CD4^+^Tm differentiation and longevity. MDSCs secrete IL‐10 and arginase products to facilitate the differentiation of Treg. Senescent neutrophils in circulation increase pro‐inflammatory activity and activate Th1 by releasing inflammatory factors during severe infections. The cooperation of MΦ with MDSC and Treg may influence other cells differentiation tendencies. Weakened ability of MΦ‐derived cytokines regulating the microenvironment may lead to immune deficiency of CD4^+^Tm. DCs, Dendritic cells; MΦ, monocyte macrophages; MDSCs, myeloid‐derived suppressor cells. The arrows represent that there are intercellular interactions.

Immunosenescence, known as immune aging, has been widely characterized since it was proposed in 1964 (Effros, 2005) and can lead to increased susceptibility to infections, impaired vaccine response, reduced cancer surveillance, chronic inflammatory infiltration, and an increased risk of age‐related diseases (Mogilenko et al., 2021; Shirakawa & Sano, 2021). In the process of aging, the immune system undergoes repeated attacks and challenges, leading to impaired functionality. Both innate immune cells (natural killer cells, monocytes/macrophages, etc.) and adaptive immune cells (T cells, B cells) experience alterations in their numbers and functions (Panda et al., 2009). Different types of immune cells are intricately intertwined and interdependent. Particularly concerning T cells, disruptions in the T cell pool and persistent inflammation contribute to premature aging of other immune cell populations (Bailin et al., 2022). Specifically observed changes include reduced CD4^+^T cell counts and proliferation capacity, impaired T cell receptor signal transduction, and cytokine production alteration (Figure 1).

CD4^+^T cells are regulated by DCs and can promote the antigen of B cell germinal center, provide key signals for B cell information transmission, induce the production of high‐affinity antibodies, and enhance the activation state and function of CD8^+^T cells and macrophages (Raphael et al., 2020). Different subsets of CD4^+^T cells, such as T helper type 1 (Th1), T helper type 2 (Th2), T helper type 17 (Th17), T follicular helper (Tfh), and regulatory T cells (Treg), secret specific cytokines that drive distinct immune responses. Especially Tregs can inhibit immune responses and help prevent autoimmune diseases (Thomas et al., 2023). Recently, clinical studies have demonstrated that the combination of low‐dose IL‐2 and methamtraxone, both known to activate and expand Tregs, exhibit a synergistic effect in ameliorating the clinical symptoms and immune disorders associated with rheumatoid arthritis (RA) (Zhang et al., 2022). Thus, the crosstalk among different subtypes of CD4^+^T cells and cytokines help to investigate the associated antiaging mechanisms and develop the treatment.

The current researches mainly focus on the roles of CD4^+^T cell subsets in the immune system during health management. However, the changes of CD4^+^T cells subsets during immunosenescence process and the inducing network among CD4^+^T cell subsets functioned in different diseases are still unclear. We aim to elucidate the intra‐ and extracellular mechanisms behind the changes of CD4^+^T cells subsets under immunosenescence and discuss the associated effects of senescent CD4^+^T cells in the development of cancer, cardiovascular and metabolic‐related diseases, and autoimmune diseases, etc., in the elderly. Moreover, we highlight the potential of therapeutic strategies that promote healthy aging and prevent aging‐related diseases by targeting CD4^+^T subsets.

## 2 ROLES OF DIFFERENT CD4 ^+^T CELLS SUBTYPES IN THE AGING PROCESS

**FIGURE 2:**
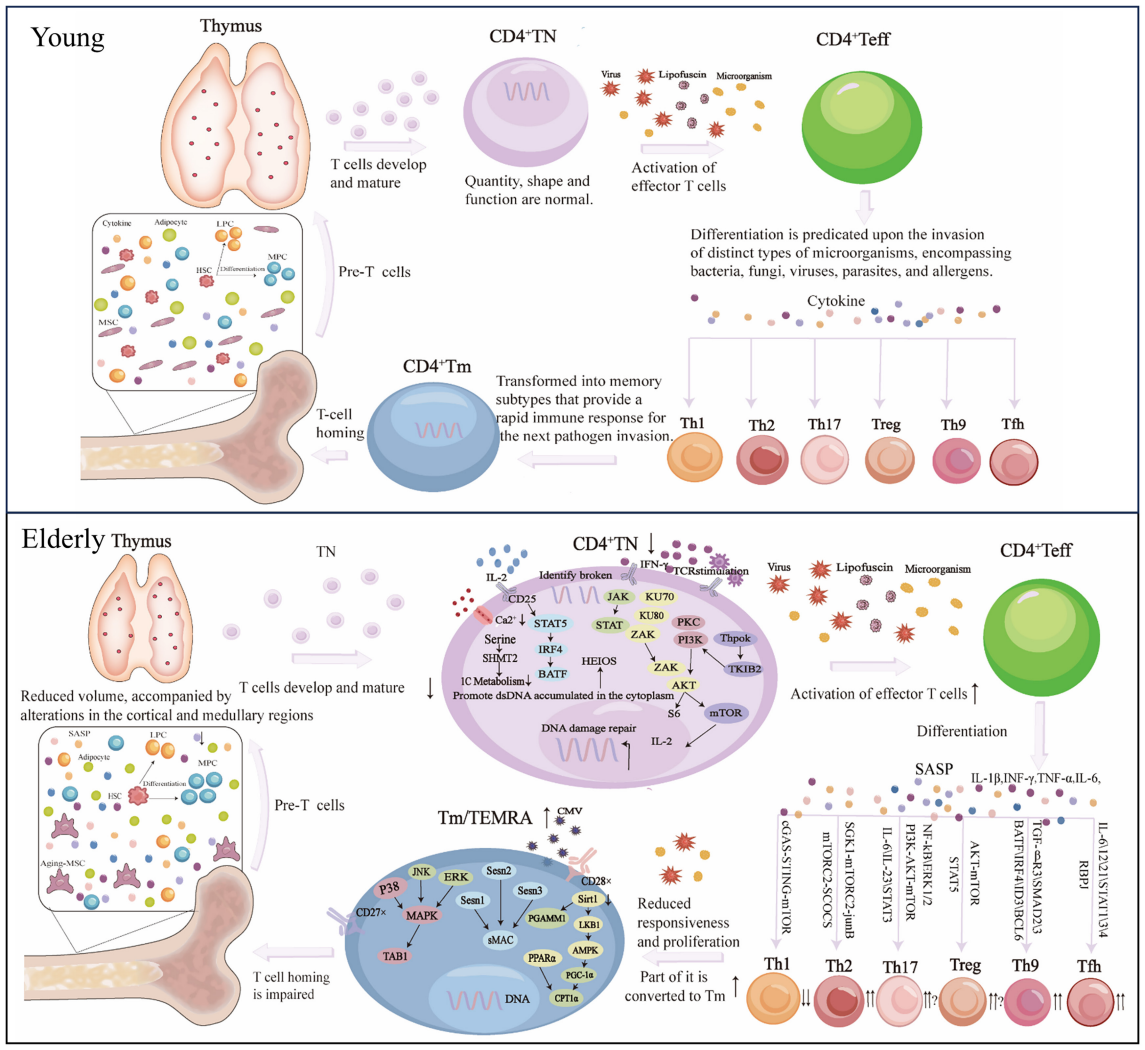
Reconfiguration of CD4^+^T cell distribution from bone marrow to peripheral compartments during immunosenescence. Aging leads to a shift in hematopoietic stem cells (HSCs) regulated by mesenchymal stem cells (MSCs), favoring myeloid cell differentiation over lymphoid cell differentiation within the bone marrow and reducing pre‐T output. In the periphery, thymus function declines, resulting in reduced maturation of CD4^+^T cells and impaired self‐renewal capacity. Due to repeated stimulation by internal and external viruses, bacteria, chronic inflammation, etc., there is a withdrawal of CD4^+^TN, differentiation of CD4^+^Teff into Th1, Th2, Th9, Th17, Tfh, and Treg. Additionally, there is an increase in the number of CD4^+^Tm. Replication senescence and impaired apoptosis of CD4^+^Tm lead to the accumulation of CD4^+^TEMRA in the bone marrow and peripherals. ↑, Increase in number; ↓, Decline in number.

The degradation in immune function can be attributed to defects in hematopoietic stem cells with age, including decreased self‐renewal potential and impaired lymphocyte generation (Pidala et al., 2020). Phenotype analysis, in vitro T lymphocyte differentiation assay, and gene expression profile of hematopoietic progenitor cells in elderly subjects, combinedly demonstrated that lymphocyte generation and active cell circulation of hematopoietic progenitor cells are impaired (Kuranda et al., 2011). Several key characteristics and considerations related to CD4^+^T cells in aging are originated from the process of emergence to differentiation (Raphael et al., 2020). For instance, the metabolic reprogramming of CD4^+^T cells participate in changes of different subtypes and functional alterations in the context of immunosenescence (Han et al., 2023) (Figure 2).

### 2.1 Decrease of Naïve CD4 ^+^T cells

Naïve CD4^+^T cells (CD4^+^TN) are an important marker during immunosenescence because of their decline with age and functional alterations (Dozmorov et al., 2017; Padilha et al., 2023). The reduction in CD4^+^TN limits the diversity of the T cell receptor (TCR) repertoire and can impair the ability of effective immune responses to new pathogens or vaccine antigens (DaFonseca et al., 2015; Saggau et al., 2022; Shirakawa & Sano, 2022). Bone marrow and thymus function are regressed, and the generation of lymphoid progenitors is downregulated (Hu et al., 2021). The thymus involution results in the reduction of CD4^+^TN due to block in TCR gene rearrangement, decrease in self‐peptide/MHC molecules, and disruption of thymic tissue architecture. And the compensatory clonal expansion is responded in the later phase to maintain the number of CD4^+^TN and homeostasis. The peripheral selection of CD4^+^TN is mainly driven by TCR signaling and cytokines, but there might be differences in clone size and inequalities during clonal expansion.

The CD4^+^TN subsets are regulated by peripheral mechanisms. The subset of CD4^+^TN has been well‐characterized after maturation by CD31‐labeling (Silva & Sousa, 2016). The maintenance of the CD31^−^CD4^+^T cell compartment is independent of the thymus but is tightly regulated by peripheral mechanisms, including CD31^−^CD4^+^T cells experiencing more proliferation, higher levels of anti‐apoptotic BFL1/A1 expression, and lower T cell receptor excision circle (TREC) relative to CD31^+^CD4^+^T cells (Bailin et al., 2022; Kohler & Thiel, 2009). The absolute number of CD31^−^CD4^+^T decreases significantly with the increase of age and the decrease of TREC (Hu et al., 2020; Muyayalo et al., 2023). IL‐7 is the main driving factor for peripheral expansion of CD31^+^CD4^+^T, and the expression of CD31 is maintained in a PI3K‐dependent manner. However, IL‐7 drive of peripheral proliferation requires sustained thymus activity (Silva et al., 2017; Takada et al., 2022).

The untimely static withdrawal of the subgroup affects the CD4^+^TN stability and functionality. The quiescent state of CD4^+^TN has low metabolic, transcriptional, and translational activities. Tribbles homolog 2 (TRIB2) is an active regulator of Protein Kinase B (AKT) (Richmond & Keeshan, 2020), and highly expressed in CD4^+^TN and regulated by the transcription factors. For example, T‐helper‐inducing POZ/Krueppel‐like factor (ThPOK) and recombinant Runt Related Transcription Factor 2 (RUNX3) are highly expressed in CD4^+^TN. In the elderly, ThPOK and TRIB2 expression decreases, which results in increased levels of IL‐7‐induced AKT phosphorylation, phosphatidylinositol 3‐kinase (PI3K) hyperactivation, and CD4^+^TN static exit (Cao et al., 2023). Rapamycin complex 1 (mTORC1) is one of the central regulators of quiescence exit. The activation of mTORC1 and AKT phosphorylation drives the increased activity of PI3K (Dibble & Cantley, 2015). The inactivation of mTORC1, such as the regulatory protein RAPTOR, and loss of RHEB (a small GTPase), inhibits PI3K signaling and prompt cells to enforce quiescence (Cui et al., 2023). HELIOS, a transcriptional repressor of T lymphocytes, is reduced in CD4^+^TN from older age groups, thus affecting the level of activation of the pSTAT5/IRF4/BATF transcription factor network (Zhang et al., 2023). Loss of HELIOS also disturbs CD4^+^TN quiescence, promotes their differentiation into tissue‐aggressive T cells, and enhances signal transducer and activator of transcription 5 (STAT5) phosphorylation, which in turn results in an inflammatory microenvironment with autoimmune inflammation and aging (Lindahl et al., 2016). In addition, the KU complex, a DNA‐dependent protein kinase (DNA‐PK) regulatory subunit, mediates DNA damage repair in the nucleus together with the catalytic subunit (DNA‐PKCs) (Walker et al., 2001). The KU complex is expressed in the cytoplasm and recognize the cytoplasmic DNA accumulated by CD4^+^TN in elderly individuals. In terms of enhancing cell proliferation and activation, KU80 and KU70 can bind better to cytoplasmic DNA to promote DNA PKCs recruitment and increase the phosphorylation level of the kinase ZAK, and activate the AKT–mTOR pathway, thereby affecting the quiescent state of CD4^+^TN and promoting cell proliferation and activation (Li et al., 2018; Wang et al., 2021).

Metabolic dysfunction results in impaired proliferation and survival of CD4^+^TN. With aging, the one carbon metabolism defect reduce the biosynthesis of purine and thymidine, which are necessary for the proliferation and survival of CD4^+^TN (Geltink et al., 2018; Ron‐Harel et al., 2018; SeongJun et al., 2022). Furthermore, the entry of Ca^2+^ into the mitochondrial matrix enhances the activity of proteins associated with the tricarboxylic acid cycle (TCA), thereby promoting oxidative phosphorylation and ATP production. In the elderly, Ca^2+^ influx upon TCR stimulation reduces in CD4^+^TN, resulting in diminished calcium‐mediated signal transduction, decreased efficiency in ATP production coupling, impaired electron transport chain function, and reduced ATP production. In the elderly, during primary activation of CD4^+^TN, the ATP is not sufficient and then the signal transduction defects and biomass synthesis impaired, which not only significantly diminish clonal expansion but also impair early cell growth (Ron‐Harel et al., 2014). Besides, the increased DNA damage and increased CD4^+^TN death anaerobic glycolysis are induced by the low efficiency of energy metabolism (Mattoo et al., 2009). The lymphocyte activation gene 3 (LAG‐3) expression in CD4^+^TN, is negative to adjust the biosynthesis and metabolism of mitochondria and regulate the steady expansion and static. Compared with the original T cells, LAG‐3 knockout CD4^+^TN exhibit greater glycolytic capacity upon activation and promote CD4^+^TN homeostasis expansion and metabolism after adoptive transfer (Previte et al., 2019). The increased LAG‐3 expression may also affect the steady‐state expansion and quiescence of elderly CD4^+^TN by regulating the metabolic level.

Lifelong exposure to environmental antigens, such as continuous stress, obesity, pathogens, and the microbiome, among others, leads to a decrease and even depletion of the CD4^+^TN subset (DaFonseca et al., 2015). For example, in the context of obesity, serum levels of adiponectin are decreased in vivo and decrease T cell proliferation and IL‐2 production in vitro (Padilha et al., 2023; Shirakawa & Sano, 2022).

### 2.2 Abnormal differentiation of effector CD4 ^+^T cells

Senescent cells and their induced inflammation, for example, senescence‐associated secretory phenotype (SASP), contribute to an aging microenvironment, which in turn affects CD4^+^TN proliferation and differentiation. The differentiation trend of effector CD4^+^T cells (CD4^+^Teff) is also an important expression during immunosenescence (Merino et al., 2021; Palatella et al., 2022). In many inflammatory diseases, the imbalance of Treg/Th17 plays an irreplaceable role (Elyahu et al., 2019), while the balance status of Treg/Th17, as well as the suppressive role of Treg for Th17, in healthy elderly and centenarians has been debated (van der Geest et al., 2014). Th17 is involved in mucosal immunity, while Tfh supports antibody production in lymphoid tissues (Song et al., 2022). Excessive Th17 responses can lead to chronic inflammation and autoimmune disorders, while impaired Treg function results in inadequate suppression of self‐reactive T cells (Bacher et al., 2019; Bharath et al., 2020; Harrison et al., 2020). Prolonged antigenic stimulation can drive the differentiation of CD4^+^T cells in the elderly towards more differentiated and functionally exhausted phenotypes, compromising their ability to mount effective immune responses.

Th1 can inhibit pathogens and tumor cells in vivo, while Th2 triggers an inflammatory response through the production of cytokines such as IL‐4 and IL‐5 (Kokubo et al., 2022). During aging, the Th1 count declines, the Th2 count rises (Zuluaga et al., 2020), and the Th1/Th2 ratio is imbalanced (Picón et al., 2021; Watson et al., 2005). The innate stimulator of interferon genes (STING) signaling in CD4^+^T cells could promote Th1 differentiation via target of rapamycin (mTOR) signaling (Benoit‐Lizon et al., 2022). cCGAs‐STING signaling could be accessing to trigger inflammatory responses and inhibit cell proliferation and apoptosis (Hou et al., 2021; Lv et al., 2022; Sladitschek‐Martens et al., 2022), which is an important signal driving the progression of senescence. mTOR complex 2 (mTORC2) could regulate the differentiation of CD4^+^TN into a Th2 phenotype, and TORC2 expression is elevated in senescent CD4^+^T cells (Delgoffe et al., 2009, 2011). Acylglycerol kinase is an mTORC2 dependent regulator that promotes Th2 phenotype differentiation by negatively regulating the degradation of the transcription factor recombinant Jun B Proto Oncogene mediated by Nedd4‐2 (E3 ligase) (Heikamp et al., 2014). In addition, mTORC2 can regulate Th2 differentiation by inhibiting suppressor of cytokine signaling (Pandit et al., 2022), indicating that mTORC2 is one of the important driving forces driving the imbalance of Th1/Th2 ratio.

Th9 is involved in the pathogenesis of allergic asthma, cancer, and other diseases. In the elderly, CD4^+^TN has a greater propensity to differentiate into Th9 (Hu et al., 2019). Upon activation, CD4^+^TN can upregulate TGFβ expression of R3, which promotes TGFβR2 versus TGFβR1 binding, phosphorylation of downstream Smad2/3, and decreases CD4^+^TN in response to TGFβ response threshold of the signal. The network of transcription factors altered in CD4^+^TN, such as increased expression of BATF, IRF4, decreased expression of ID3 and BCL6, and the altered transcription factors, synergistically drive Th9 differentiation (Kaplan, 2017; Pei et al., 2021).

Th17 mediates pro‐inflammatory responses in vivo, while Treg promotes anti‐inflammatory cytokine production. The elevated Th17/Treg ratio has been found in inflammatory versus autoimmune diseases during aging. The ratio of Th17/Treg is higher in the elderly, but turn lower in long‐lived centenarians, which may be important in attenuating chronic inflammation as well as prolonging lifespan (Zhou et al., 2022). Th17 differentiation could be activated by the PI3K‐AKT–mTOR pathway and STAT3 signaling, whereas STAT5 signaling, low AKT–mTOR signaling, and high TGFβ signaling favor Treg differentiation (DiToro et al., 2020; Zhou et al., 2022). Age‐related metabolic changes can also modulate the equilibrium of Th17/Treg ratio (Bishop et al., 2024). For instance, in autoimmune diseases such as RA, IGF receptor signaling activates the AKT–mTOR pathway and enhances aerobic glycolysis, thereby favoring Th17 differentiation over Treg differentiation (DiToro et al., 2020). There are limited studies about the metabolic reprogramming of senescent CD4^+^TN differentiation and need further exploration.

Tfh subsets differentiate from CD4^+^T cells exposed to a special cytokine milieu and are indispensable for immunoglobulin production while providing cognate help to B cells, influencing GC formation and function (Silva‐Cayetano et al., 2023; Varricchi et al., 2020). Tfh differentiation increases during aging, and aging affects early Tfh differentiation. The accumulation of pre‐Tfh in elderly individuals owing to altered TCR signaling results in increased expression of the notch‐related transcription factor RBPJ and promotes enhanced pre‐Tfh differentiation (Webb et al., 2021). In addition, in the senescent microenvironment, IL‐12, IL‐6, and IL‐21 are expressed by STAT4, STAT3, and STAT1 to induce BCL6 and Tox2 expression, thereby influencing Tfh differentiation (Herati et al., 2021).

### 2.3 Functional disturbances of memory CD4 ^+^T cells

The memory CD4^+^T cells (CD4^+^Tm) phenotype is acquired and accumulates with age owing to accelerated homeostatic proliferation induced by lymphopenia, as well as in response to cytokines (Hu et al., 2020). Memory an altered relative size of the nucleus, which confers increased cell stiffness, reduced cell migration (González‐Bermúdez et al., 2022; Hu et al., 2020, 2022), and impaired immunity in the elderly. Due to the long‐term repeated stimulation of antigen, the number of CD4^+^Tm in the elderly increases (Appay & Sauce, 2014). The lost expression of costimulatory molecule CD27 and CD28, and upregulated expression of killer cell lectin‐like receptor subfamily g (KLRG‐1) and CD57, result in forming and accumulating the terminally differentiated CD4^+^T cells subtype (CD4^+^TEMRA) (Huang et al., 2023). CD4^+^TEMRA reduce proliferative capacity, decrease diversity of the antigen recognition repertoire and reduced T cells activation. The p38 MAP kinase in CD4^+^T cells can be activated by MAPK signaling or the alternative TCR pathway to reconstitute telomerase activity. CD4^+^TEMRA engage the metabolic sensor AMPK to trigger p38 recruitment to the scaffold protein TAB1, which results in p38 autophosphorylation and inhibits telomerase activity and cells proliferation potential (Henson et al., 2015; Lanna et al., 2014). p38, ERK, and JNK are localized within a separate inhibitory signaling complex that also contains the stress proteins Sestrins (mammalian products of Sesn1, Sesn2 and Sesn3) in CD4^+^TEMRA containing AMPK. In addition, the subunits of two other MAPKs are also known to regulate CD4^+^TEMRA function. Both clinical and animal analysis indicated significant increase of the Sestrins‐MAP kinase activated complex (sMAC) in older groups (Lanna et al., 2017; Laphanuwat et al., 2023). Blocking of sMAC not only elevates antigen‐specific proliferation of CD4^+^TEMRA but also enhances the response to influenza vaccination in elderly mice. The accumulation of CD4^+^TEMRA in the elderly is closely correlated to the occurrence and development of several diseases, but the excessive elimination of the CD4^+^TEMRA burden may disrupt tissue homeostasis.

The metabolic reprogramming of CD4^+^Tm exhibits intracellular signal disturbance and impaired cell function, which not only promotes the secretion of aging‐related inflammatory factors but also diminishes the elderly population's ability to combat viral infections and reduces the efficacy of vaccines (Liu et al., 2023; Strickland et al., 2023). As CD4^+^Tm aging, the respiratory capacity, mitochondrial content, and intracellular ROS generation are all increased in response to TCR stimulation. However, the unchanged glucose uptake and cell ATP levels cause the secretion of pro‐inflammatory cytokines such as IFN‐γ and IP‐10 (Chen et al., 2022). In addition, expression of SIRT1 (an important regulator of fatty acid metabolism) is decreased in CD4^+^TEMRA, which inhibit the activation of AMPK by SIRT1‐mediated LKB1 deacetylation and AMPK‐mediated PGC‐1α phosphorylation. In glucose and fatty acid metabolism, phosphorylated PGC‐1α interacts with PPARα to induce reduced transcription of CPT1a and altered CD4^+^Tm survival in post‐infection senescence (Yanes et al., 2019). Moreover, SIRT1 alleviates glycolysis through the deacetylation of phosphoglycerate mutantase‐1, the reduced expression of which could lead to the increased glycolysis and decreased oxidative phosphorylation in CD4^+^TEMRA. Decreased energy level in CD4^+^TEMRA could promote the activation of AMPK pathway, affect the binding of telomerase reverse transcriptase gene and transforming growth factor activated protein kinase binding protein 1 (TAB1), and induce downstream p38 autophosphorylation (Han et al., 2023; Lanna et al., 2014), which further accelerate CD4^+^T cells senescence and DNA damage.

## 3 THE IMMUNOSENESCENT CD4 ^+^T CELLS ACCELERATE AGE‐RELATED DISEASES

**FIGURE 3:**
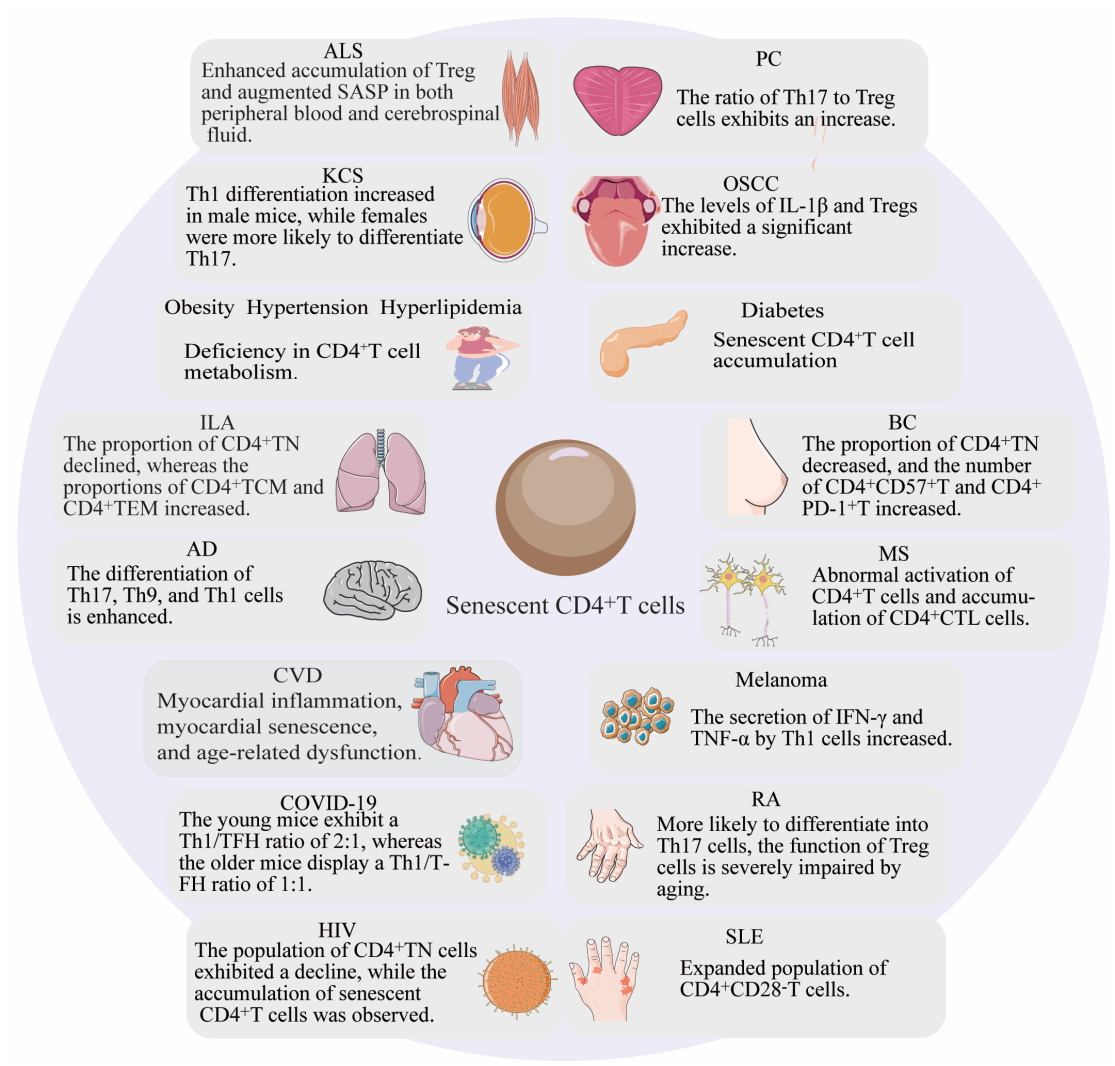
The crucial role of CD4^+^T cells in age‐related diseases. AD, Alzheimer disease; ALS, Amyotrophic lateral sclerosis; BC, Breast cancer; COVID‐19, Corona virus disease 2019; CVD, Cerebrovascular disease; HIV, Human immunodeficiency virus; ILA, Interstitial lung abnormality; KCS, Malignant keratoconjunctivitis; MS, Multiple sclerosis; OSCC, Oral squamous cells carcinoma; PC, Prostate cancer; RA, Rheumatoid arthritis; SLE, Systemic lupus erythematosus.

Elderly patients experience significantly greater disease severity and mortality compared to young patients. CD4^+^T cells play a crucial role in protective immunity among the elderly population, making them a potential target for improving strategies against immunosenescence (Swain et al., 2005). Although specific treatments for immunosenescent CD4^+^T cells are still under investigation, modulation of CD4^+^T cells holds promise for addressing the following diseases (Figure 3).

### 3.1 Cancers

Immunosenescence constitutes a pivotal risk factor for the occurrence and progression of diverse malignancies (López‐Otín et al., 2023; Pérez et al., 2022). Several tumors, such as melanoma, PC, etc., interplay between CD4^+^T cells and other age‐related malignancies in the elderly (Borst et al., 2018; Oliveira et al., 2022; Speiser et al., 2023). Many studies have substantiated the significance of CD4^+^T cells recognition of tumor antigens in tumorigenesis, response to immunotherapy, and prognosis of individuals with neoplasms (Hirschhorn et al., 2023; Su et al., 2017).

Melanoma is an immunological malignancy that poses a novel challenge for immunosenescent elderly individuals (Spiliopoulou et al., 2023). The intrinsic mechanism of melanoma involves the alleviation of CD4 inhibitory effect, leading to the secretion of IFN‐γ and TNF‐α by stimulated Th1 (Kruse et al., 2023). These two cytokines can collectively induce tumor cell senescence, escape from unlimited proliferation after senescence, cause cell cycle arrest, and promote SASP secretion. Although SASP can lead to an inflammatory microenvironment, short‐term moderate inflammation can therapeutically inhibit tumor growth (Homann et al., 2022). Anti‐PD‐L1 therapy‐induced CD4^+^T cell‐derived IL‐21‐CXCL13 accumulation in organs is associated with immune‐related adverse events in elderly mice harboring melanoma (Granier et al., 2021; Tsukamoto et al., 2022). To effectively manage melanoma in elderly patients, it is crucial to target CD4^+^T cells and focus on the cytokines and downstream signals they secrete.

PC is one of the most prevalent malignancies in elderly males (Chang et al., 2014). The older tumor‐bearing mice exhibit an increased Th17/Treg ratio compared to younger mice. Besides, age‐related factors associated with Th17 (IL‐17A, IL‐17F, and IL‐22) have been found to activate NF‐κB and ERK1/2 signaling pathways, thereby promoting tumor cell growth (Liu et al., 2020). The Treg number is elevated within samples from PC patients. The accumulation of Treg and Th17 appears to be a significant risk factor for prostate disease and PC in response to *Propionibacterium acnes* infection (Radej et al., 2022). Thus, emphasizing the equilibrium between Treg and Th17 as well as their accumulation in aged individuals holds great significance for effective prevention strategies and targeted therapeutic approaches against PC.

The incidence of OSCC in the elderly is gradually increasing (Pai et al., 2021). The tongues of aged mice with oral cancer exhibited a significant increase in the abundance of IL‐1β and Treg. Their partial absence leads to reduced tumor burden (Bhaskaran et al., 2021). Furthermore, there is accumulation of Treg in elderly patients with lung cancer, which enhances their susceptibility to OSCC (Hou et al., 2017). Tregs possess the ability to suppress anti‐tumor immune responses, thereby diminishing the efficacy of cancer immunotherapy. Manipulating the generation and differentiation of Tregs and enhancing their depletion holds promise for novel approaches to prevent and treat various cancers.

An increase in the proportion of senescent CD4^+^T cells is observed in the tumors and lymph nodes of BC patients. High gene expression levels of CD4, KLG‐1, and CD57 are associated with improved overall survival in BC patients (Ramello et al., 2021). Besides, the percentage of CD4^+^TN decreases in BC patients, whereas the numbers of CD4^+^CD57^+^T cells and CD4^+^PD‐1^+^T cells increases. However, following complete removal of the tumor mass, there is a switch in the subtype of CD4^+^T cells, which alters the patient's immune depletion and immunosenescent status (Lu et al., 2023).

Attention to CD4^+^T cells in the context of immune aging plays a notable role in predicting cancer occurrence, inhibiting cancer development, enhancing the efficacy of cancer treatment, mitigating its side effects, and prognosticating outcomes for cancer patients. This holds immense significance for reducing cancer incidence and mortality among the elderly population while improving the management and treatment of malignant diseases.

### 3.2 Cardiovascular and metabolic‐related diseases

Aging is closely related to metabolic imbalance in the body, involving various aspects such as carbohydrate metabolism, steroid metabolism, and purine metabolism. CD4^+^T cells play an indispensable role in the aging changes of various cardiovascular and metabolic‐related diseases (Wiley & Campisi, 2021).

Diabetes can lead to multiple organ damage in elderly patients and further aggravate the accumulation of senescent cells in the body (Palmer et al., 2019). Dynamic interactions between CD4^+^CD25^+^ inhibitory T cells and CD4^+^CD25^−^ pathogenic T cells are associated with diabetes progression in non‐obese diabetes mice (Gregori et al., 2003). The senescent CD4^+^T cells accumulated in patients with type 2 diabetes and the impaired migration of these cells (Lau et al., 2019) may further exacerbate clinical symptoms and disease progression (Denroche et al., 2021). It is necessary that monitoring the changes in senescent CD4^+^T cells to track immune changes and identify potential risk factors in elderly with diabetes.

The aging immune system and cardiovascular system are intricately intertwined, significantly impacting the disease progression. The immune homeostasis caused by the proliferation and senescence of CD4^+^T cells is involved in age‐related cardiac dysfunction (Ross et al., 2018). The number of cells in mediastinal lymph nodes increases in aging hearts, and the CD4^+^T effector memory phenotype comes out (Shirakawa & Sano, 2021). The mediastinal lymph nodes in older mice are mainly composed of foxp3‐CD4^+^T cells, which is different from that in younger mice (Ramos et al., 2017). Furthermore, excessive accumulation of memory T cells and TEMRA correlates with an elevated risk of cardiovascular disease, heart failure progression, and increased cardiac‐specific mortality rates.

The incidence of hypertension is a significant risk factor in cardiovascular diseases (Egan et al., 2024). Although the proportion of pre‐menopausal women suffering from hypertension is low, the control symptoms after menopause is difficult. After angiotensin II infusion, the phosphorylation level of TLN1 (a positive regulator of Treg function), the transcription levels of Foxp3 and IL‐10 in CD4^+^T cells of postmenopausal mice decrease, and the phosphorylation level of phosphorylation sites related to ERK activity increase. CD4^+^T cell signaling plays a significant role in promoting heightened inflammation during the onset of postmenopausal hypertension. There is an increase in the total number of white blood cells in patients with refractory hypertension, primarily attributed to the elevation of CD4^+^T cells, which is accompanied by an imbalance in the Th17/Treg ratio, characterized by a significantly higher count of CD4^+^IL‐17A^+^T cells (Imiela et al., 2022).The prostaglandin D2/D‐prostaglandin receptor 1 (DP1) axis is down‐regulated in CD4^+^T cells of elderly mice with hypertension. DP1 could inhibit T‐box‐expressed‐in‐T‐cells (T‐bet) ubiquitination mediated by the NEDD4L pathway through protein kinase A/phosphorylated specific protein 1/neuroprogenitor cell expression development, thereby suppressing Th1 activity. Forced overexpression of exogenous DP1 in T cells has been shown to attenuate age‐related hypertension in mice by downregulating Th1 cytokine expression through the aforementioned mechanism (Kong et al., 2020).

Aging is accompanied with abnormal lipid metabolism, even atherosclerosis. CD4^+^TEMRA accumulate in the appendages of atherosclerotic plaques in patients and can secrete significant amounts of CCR5, CCR7, and CXCR1, promoting inflammation at the site of atherosclerotic plaques (Gaddis et al., 2021). Treg cells acquire markers of Th1, Th17, and Tfh or transition into a more memory‐like phenotype further exacerbating atherosclerosis possibly linked to pro‐inflammatory environment formation (Bazioti et al., 2023). Studies on hyperlipidemia and atherosclerosis in elderly patients can explore not only metabolism but also from CD4^+^T cells' metabolic changes among various subtypes.

Obesity can lead to systemic inflammatory infiltration in the elderly and promote the incidence of various age‐related diseases increases (Wijngaarden et al., 2021). The changes in the CD4/CD8 ratio in obese elderly individuals can serve as an important prognostic marker for immune aging (Tylutka et al., 2024), while alterations in CD4^+^TN percentage can predict insulin sensitivity in elderly obese patients, providing valuable guidance for stratified research on obesity treatment (Sbierski‐Kind et al., 2020). Future studies could focus on combating CD4^+^T cell aging, modulating mitochondrial function within senescent cells, improving metabolism, reducing inflammatory factor release, and enhancing physical well‐being among obese elderly individuals.

### 3.3 Infections

Aging individuals exhibit heightened susceptibility to infections owing to the waning functionality of their immune system. The protective role of CD4^+^T cells in pulmonary infectious diseases and during vaccination diminishes with age, rendering influenza and COVID‐19 more severe in the elderly population (Sant et al., 2018). Although the total number of CD4^+^T cells in the lungs remains unaffected by age following influenza infection, there is a significant disparity observed in Th subgroup distribution, with Th1/Tfh of 2/1 ratio prevailing in young mice while Th1/Tfh of 1/1 ratio dominates among old mice (Lorenzo et al., 2018). Notably, immune aging correlates with clinical inflammatory status as evidenced by COVID‐19 patients displaying alterations not only in absolute levels of CD4^+^T cells but also their proliferative capacity and mitochondrial function. The COVID‐19 patients tend to differentiate towards a pro‐inflammatory phenotype characterized by Th17 which may serve as drivers for cytokine storms on a large scale (De Biasi et al., 2020). It is suggested that it is necessary to enhance pathogen recognition, cytokine production, and effective immune response by promoting the production and function of CD4^+^T cells.

Depletion of a substantial number of CD4^+^T cells is one of the primary indicators of HIV‐1 infection. CD4/CD8 T cells serves as a prognostic marker for HIV infection (Chauvin & Sauce, 2022; Serrano‐Villar et al., 2022). Individuals infected with HIV exhibit reduced responsiveness of CD4^+^T cells to IL‐7 stimulation, which further impacted downstream JAK/STAT5 signal transduction and disrupted CD4^+^T cell homeostasis, thereby exacerbating both aging and disease progression (Bazdar et al., 2009). Profiling the precise correlation between immune status and aging in HIV‐1 infection could potentially enhance care provision and monitoring strategies for patients living with HIV‐1.

### 3.4 Autoimmune diseases

Diseases characterized by immune disorders and autoimmunity exhibit a higher prevalence among the elderly population. RA accelerates immunosenescence, and its pathological changes are closely associated with CD4^+^T cells (Andonian et al., 2022). Furthermore, elevated levels of IL‐6 and TGF‐β in RA induce differentiation of CD4^+^T cells into Th17, while aging significantly impairs the function of Treg, leading to their inhibitory function inactivation and Th17/Treg imbalance (a significant characteristic of RA) (Gao et al., 2022; Turcinov et al., 2023).

Disruption of homeostasis and functional defects in Th17/Treg also contribute to the progression of SLE. Senescent SLE is characterized by the expansion of CD4^+^CD28^−^T cells, which exacerbates tissue function aging and promotes overall health deterioration (Moysidou et al., 2022). However, it has not been reported whether CD4^+^T cell senescence‐related phenotypes and functional loss are implicated in the pathogenesis of SLE.

The abnormal inflammatory process of MS involves the regulation of T cells, B cells, and innate immune cells, particularly CD4^+^T cells. There is a downregulation of CTLA‐4 expression (an age‐related T‐cell co‐inhibitory receptor) in elderly patients, accompanied by an upregulation of B‐cell co‐stimulatory molecules. These alterations contribute to the aberrant activation of CD4^+^T cells and the accumulation of CD4^+^CTL. Furthermore, even among the young individuals with MS, there is a disproportionate increase in the proportion of CD4^+^Tm, indicating a persistent low‐grade inflammatory activity within the central nervous system (Zuroff et al., 2022).

Building upon previous research, it has been observed that elderly patients with autoimmune diseases exhibit abnormal accumulation of CD4^+^Tm and significant alterations in the differentiation propensity of CD4^+^Teff, which could serve as a crucial intervention point for preventing disease onset and progression. Notably, targeting the imbalance between Th17/Treg may hold promise for addressing autoimmune disorders.

### 3.5 Other chronic inflammatory diseases

Chronic inflammation is a hallmark of aging and contributes to a diverse range of age‐related diseases. Modulating the CD4^+^T cell subsets from overall system to local organ and associated cytokine profile holds promise in reducing disease risk.

Elderly individuals face a significantly heightened risk of developing lung inflammatory diseases (Laidlaw et al., 2016). ILA is accompanied by decreased lung function and increased respiratory symptoms (Sanders et al., 2021). Compared to healthy elderly subjects, the proportion of CD4^+^TN decreases in elderly individuals with ILA, while the proportions of CD4^+^TCM and CD4^+^TEM increase (Machahua et al., 2021). Furthermore, CD4^+^T cells exhibit enhanced proliferation and pro‐inflammatory capabilities, leading to elevated levels of IL‐1β, IL‐6, and IFN‐γ. The accumulation of CD4^+^TEM within the lungs cause IFN‐γ upregulation in patients with age‐related pulmonary fibrosis. Activate TGF‐β1/IL‐11/MEK/ERK signaling pathway to promote epithelial mesenchymal transition and cellular senescence in type II alveolar epithelial cells (Chen et al., 2023). Therefore, targeting IFN‐producing CD4^+^T cells along with other inflammatory factors may serve as potential therapeutic strategies for preventing age‐related respiratory diseases.

The impact of aging on inflammation exacerbates the prognosis in ALS patients (van Deursen, 2014). The elderly individuals with ALS exhibits an age‐related phenotype of CD4^+^T cells along with an accumulation of Treg cells, which is associated with decreased survival rates (Yildiz et al., 2023). The depletion of selective CD4^+^T lymphocytes may be potential therapeutic strategy for managing ALS.

In the peripheral blood of elderly individuals with AD, there has been an observed increase in the differentiation of CD4^+^Teff into Th17, Th9, and Th1 subsets (Machhi et al., 2021). Notably, IL‐17 serves as the primary secretory factor for Th17 and can infiltrate the brain, leading to disruption of the blood–brain barrier and subsequent cognitive impairment along with synaptic defects. However, conflicting evidence exists regarding the role of Th1 in AD patients (Brigas et al., 2021). Additionally, obesity elevates levels of TEM, Treg, and N‐acetylneuraminic acid metabolite in AD mice models (Suzzi et al., 2023). Consequently, it becomes imperative to elucidate distinct functions associated with different CD4^+^Teff subtypes to mitigate immune deficiencies present in AD patients.

The increasing incidence of KCS is closely associated with immunosenescence, particularly in old women (de Benedictis et al., 2000). There is a higher inclination towards Th1 differentiation for CD4^+^Teff in male mice, while a greater tendency towards Th17 differentiation in female mice, with the disruption of the ocular barrier and increased expression of MMPs by IL‐17A (McClellan et al., 2014). An elevated infiltration of CD4^+^T cells is observed in the conjunctiva of older mice, which promoted increased levels of IFN‐γ and IL‐17 mRNA, exacerbating the ocular inflammatory response (Trujillo‐Vargas et al., 2022). By manipulating CD4^+^Teff differentiation, it is possible to alter the immune landscape within the lacrimal gland to delay ocular aging processes and effectively rejuvenate vision among elderly individuals.

Above, targeted therapy of CD4^+^T cells for inflammatory diseases in the elderly can focus on the specific mechanism of action of different subtypes of CD4^+^Teff and formulate personalized treatment strategies according to the specificity of the disease.

## 4 STRATEGIES TO DELAY IMMUNOSENESCENCE BY REGULATING CD4 ^+^T CELLS

The development of targeted treatments specifically altering CD4^+^T cells in aging individuals is an ongoing area of research on the treatment of specific diseases and age‐related conditions (Table 1). Initial efficacy has been demonstrated in the regulation of CD4^+^T cells to improve aging through interventions such as vaccines, immunotherapy, drug therapy, cell therapy, and lifestyle modifications.

**TABLE 1:** Treatments in specific disease by delay immunosenescence via CD4^+^T cells.

Diseases	Subject or model	Treatment	Changes in CD4^+^T cells			Changes in cytokines	Reference
			CD4^+^TN	CD4^+^Teff	CD4^+^Tm/TEMRA		
Influenza	60–80 elderly	High Dose Influenza and Adjuvanted Influenza Vaccine		Regulating Th1/Th2 cells differentiation		IL‐17↑	Haralambieva et al. (2022)
	Individuals aged 65 and above	AS03 adjuvant vaccine		Stimulating the influenza specific CD4^+^T cells response		IL‐2↑, IFN‐γ↑	Couch et al. (2014)
COVID‐19	60–84 elderly	Injection of NVX‐CoV2373 (adjuvanted recombinant full‐length SARS‐CoV‐2 spike protein vaccine)		Tfh↑, Th1↑		IFN‐γ↑, TNF‐α↑, and IL‐2↑	Rydyznski Moderbacher et al. (2022)
Age related joint injuries	72‐week‐old male C57BL/6 mice	IL‐17 neutralizing antibody	Transient increase in peripheral CD4^+^TN		Not diminishing the diversity of TCR repertoire		Faust et al. (2020)
Acute lymphoblastic leukemia	Aged mice (≥24 months)	rIL‐37^+^CAR‐T cells therapy	PD‐1 expression↓ on CD4^+^TN			IL‐2↑, IFN‐γ↑, PD‐1↓	(Hamilton et al. (2021)
Natural aging	high‐fat diet (HFD)‐induced obese C57BL/6J mice	CD153 vaccine			number↓	IgG2↑	Yoshida et al. (2020)
	Rhesus Macaques (>20 years)	IL‐7 Therapy	number↑				Okoye et al. (2015)
	18‐month‐old wild‐type mice	Exercise and caloric restriction	Reversing the effects of immune senescence	Reversing the consequences of immune senescence		PD‐1↓, Tim‐3↓, KLRG1↓, NR4A1↑, TOX↑	Asami et al. (2022)
	Yoga (46.0 ± 9.4) Non‐yoga (41.8 ± 9.7)	Yoga exercise		Reestablishing and sustaining a stable equilibrium between Th17 /Treg		RORγt↓, IL‐17↓, IL‐6↓, CXCL2↓, CXCR2↓, FoxP3↑, TGF‐β↑	Gautam et al. (2023)

### 4.1 Vaccination

Influenza vaccination is considered as the primary intervention for safeguarding the elderly population and mitigating complications (Lefebvre et al., 2016). The administration of high dose influenza vaccine and adjuvanted influenza vaccine could modulate the expression of transcriptional activity/function‐related genes and proteins in CD4^+^T cells. The efficacy of AS03 adjuvant vaccine in preventing H3N2‐associated influenza A has been confirmed by clinical trial, which also demonstrates the induction of a multifunctional CD4^+^T cell response in the elderly population (Couch et al., 2014). In addition, age‐related decline in the quality of vaccine‐induced immune response against SARS‐CoV‐2 can be attributed to inherent defects in the CD4^+^T cell pool. Within 7 days of initial administration of NVX‐CoV2373 (a recombinant full‐length SARS‐CoV‐2 spike protein vaccine with adjuvant), circulating follicular T helper cells and Th1 cells could be detected (Rydyznski Moderbacher et al., 2022; Saggau et al., 2022). The robust generation of CD4^+^T cells serves as an effective adjuvant for optimal vaccine efficacy. CD153 vaccine reduces the number of senescent T cells in mice through the production of mouse IgG2 antibodies upon administration (van Deursen, 2014; Yoshida et al., 2020). The phenotype and function of CD4^+^T cells can limit the antibody titer and immunogenicity observed in elderly individuals following vaccination. Future efforts should not only explore adjuvants and formulations for augmenting vaccine immunogenicity but also seek more effective strategies for improving vaccine efficacy.

### 4.2 Immune modulators

Immunomodulatory therapy has been used to enhance the CD4^+^T cell response in the elderly. For instance, in a mouse model of joint injury, aging‐related Th17 immune response is augmented, and intra‐articular administration of IL‐17 neutralizing antibodies can modulate Wnt signaling, mitigate joint degradation, and reduce the expression of senescence markers (Faust et al., 2020). Additionally, recombinant interleukin‐7 (rsIL‐7), an immune agent, has shown transient efficacy in increasing CD4^+^TN and CD4^+^Tm counts among elderly Yokogawa patients by influencing their adaptive immune response (Okoye et al., 2015; Pandit et al., 2023). The involvement of JAK/STAT signaling pathway appears to be implicated in rsIL‐7 regulation. Recombinant interleukin‐37 (rIL‐37) can restore the gene expression profile of senescent CD4^+^T cells and reduce the levels of immunosuppressive proteins Tim‐3 and TIGIT in senescent T cells, thereby effectively modulating the immune microenvironment (Hamilton et al., 2021). Although the immunomodulators have exhibited significant potential in augmenting the immune response of CD4^+^T cells in fundamental investigations, further investigation is warranted to comprehensively ascertain whether treatment with immunomodulators alone or in conjunction with other immunostimulators can effectively reverse immunosenescence and mitigate the deleterious consequences of therapy in the geriatric population.

### 4.3 Cellular therapies

Adoptive transfer of in vitro amplified or genetically modified CD4^+^T cells is an emerging strategy that holds promise for restoring immune function in the context of immune aging (Kofler et al., 2011). CAR‐T cells generated from TN and TCM exhibit strong cytotoxicity due to cytokine production by CD4^+^T cells, and are more effective compared to TEM CAR‐T cells (Cooke et al., 2020; Yi et al., 2022). IL‐37 possesses the ability to directly inhibit TNF‐α signaling and downregulate PD‐1 surface expression in senescent CD4^+^T cells to enhance the efficacy of CAR‐T cells therapy in elderly individuals. Administration of rIL‐37 in aged mice could reduce TNF‐α signaling, decrease PD‐1 surface expression on initial CD4^+^T cells, and prevent elevated PD‐1 expression on aged CAR‐T cells (Hamilton et al., 2021). CAR‐T cells therapy has been proved to be successful in the immunotherapy of hematological malignancies as well as solid tumors (Coppola et al., 2020). Current cell preparations exhibit a high recurrence rate and fall short of meeting expectations in terms of therapeutic efficacy. Future endeavors should focus on conducting comprehensive fundamental and clinical research to unveil alternative mechanisms underlying immune evasion.

### 4.4 Pharmacological interventions

Several pharmacological interventions have been investigated to modulate the functionality of CD4^+^T cells and ameliorate the consequences associated with immune senescence (Table 2). For instance, inhibitors targeting the mTOR pathway have exhibited promising potential in clinical research for reinstating CD4^+^T cell responsiveness and fostering immune homeostasis. Studies have demonstrated that the administration of anti‐allergy drugs (dasatinib and quercetin) or immunotoxin (anti‐HCD2‐SAP) in mice can induce CD4^+^T cells differentiation towards a more youthful phenotype, enhancement of immune aging in elderly mice (Ghamar Talepoor et al., 2021). Plastoquino‐nyl decyltriphenyl phosphonium (SkQ1) can increase the number of CD4^+^T cells and enhances the ratio of CD4^+^T/CD8^+^T cells within the thymus, suggesting its potential for enhancing immune function (Obukhova et al., 2009). Metformin can rectify autophagy defects and mitochondrial dysfunction in CD4^+^T cells of elderly individuals (Bharath et al., 2020). Long‐term administration of icariin in AD mice could significantly attenuate the differentiation propensity towards Th1 and Th17, mitigated immune‐inflammatory responses mediated by CD4^+^T cells (Zhu et al., 2019). Gallic acid and resveratrol can increase the number of CD4^+^T cells in thymus or spleen of aging mice induced by D‐gal and alleviate the decline of immune system (Guo et al., 2020; Wei et al., 2021). Small molecular compounds such as curcumin, 17‐estradiol, and genistein have regulatory effects on thymus aging (Selvaraj et al., 2005; Wei et al., 2021), making them potential drugs for modulating CD4^+^T cells to improve aging.

**TABLE 2:** Pharmacotherapy benefits to delay immunosenescence via CD4^+^T cells.

Pharmacological agent	Subject or model	Health benefit	Specific mechanism of action	Reference
mTOR inhibitor (RAD001)	Elderly individuals	Promoting a healthy immune system	PD‐1^+^CD4^+^T↓, restoring the immune response of CD4^+^T cells	Mannick et al. (2014)
Dasatinib and quercetin	Older Mice	Improving the immune senescence of aged mice	Expressing FoxP3 Th cells↓, T cell differentiation reverted to a younger phenotype	Lorenzo et al. (2022)
Anti‐HCD2‐SAP	p16hCD2 mice	Improving the antiviral clearance mechanism	p16 expression↓, CD4^+^Tm↓, CD4^+^TN↑	Sugiyama et al. (2023)
SkQ1	Elderly individuals	Delaying thymic decline and enhance immune function	CD4^+^T cells and CD4^+^/CD8^+^T cells ratio↑	Obukhova et al. (2009)
Metformin	Elderly individuals	Improving the inflammatory state of aging population	Oxidative phosphorylation of CD4^+^T cells↑, non‐mitochondrial glycolysis, ameliorates↑STAT3 levels↓, Th17‐related inflammation↓	Bharath et al. (2020)
Icariin	D‐galactose‐induced senescence in mice	Improving cognitive deficits in mice	Regulating the differentiation of Th1, Th17, and Treg cells, inflammatory factors↓	Zhu et al. (2019)
Gallic acid	Ovariectomized female mice	Alleviating immune system decline	CD4^+^T cells↑, restoring the balance of CD4^+^/CD8^+^ T cells	Guo et al. (2020)
Resveratrol	D‐galactose‐induced senescence in mice	Increasing CD4^+^T count and IL‐2 levels in the spleen	Thymus function ↑, IL‐2↑	Wei et al. (2021)

### 4.5 Lifestyle modifications

Lifestyle factors such as regular exercise, balanced nutrition, and stress management have been associated with improvements in immune function. For instance, voluntary regular exercise and long‐term caloric restriction can effectively reduce immune senescence in elderly mice by reversing the effects of immune senescence on CD4^+^TN and CD4^+^Teff subsets (Asami et al., 2022; Martin et al., 2013). Low‐protein diet intake could reverse aging changes of CD4^+^T cells in the spleen (Le Couteur et al., 2015). RA patients can maintain Th17/Treg homeostatic balance through yoga exercises thereby reducing T‐cell aging rate while improving RA severity (Gautam et al., 2023). Promoting healthy lifestyles among elderly populations is helpful in enhancing body resilience stability leading to better quality life.

## 5 CONCLUSIONS AND FUTURE PROSPECTS

As individuals age, significant alterations occur in the internal and external milieu of CD4^+^T cells. These changes encompass reduced CD4^+^TN levels, thymic hypofunction, peripheral mechanism regulation, untimely quiescent withdrawal, and persistent environmental antigen stimulation. The interplay between the in vivo microenvironment and the aging immune system is intricately linked, resulting in a decline in CD4^+^Teff proliferation capacity, alterations in differentiation patterns, imbalances in Th1/Th2 ratio, changes in Th17/Treg ratio, among others. Repeated antigenic stimulation, accelerated homeostasis, and delayed clearance lead to impaired mitochondrial respiration, reduced functionality, accumulation of memory subpopulations with autophagy deficits, loss of CD27 and CD28 surface molecule expression, increased production of cytotoxic molecules, and elevated levels of CD4^+^TEMRA. Enhancing the function of CD4^+^T cell phenotype and targeted depletion thereof represents a crucial approach for improving the immune microenvironment in elderly individuals. Future exploration can focus on the mitochondrial dysfunction, metabolic reprogramming, genetic and epigenetic changes, protein homeostasis imbalance, autophagy defects, loss of cellular plasticity, and reduction of TCR pool in aging CD4^+^T cells to clarify the nature of changes in different subtypes of CD4^+^T cells under immune aging. More attention should be paid to mutual influence and interaction in the process of CD4^+^T cell aging, which are necessary to reverse both multi‐organ senescence and immune senescence. According to the extracted and interconnected network depicted in Figures 1 and 2, it is evident that CD4^+^T cells serve as the central hub, not only influencing other immune cell populations but also orchestrating changes within internal subsets and related signaling pathways. Through systematic summarization of these findings, we investigate a profound comprehension of the pivotal role, intricate nature, and challenges associated with CD4^+^T cells in immune aging. The underlying mechanisms involved are elucidated and analyzed. It is our aspiration that this comprehensive understanding can contribute to clinical practice by bridging the gap between clinical applications and fundamental research, thereby facilitating the development of enhanced preventive measures, treatments, and interventions aimed at averting health deterioration while promoting healthy aging among elderly individuals.

In terms of treatment, although the current treatment has achieved good results from vaccination, immune modulators, cell therapy, drug therapy, and life intervention, there are still some outstanding problems in this field that need to be explored. Firstly, vaccine research is mainly limited to the direction of anti‐influenza infection, and it also needs to be extended to the regulation of the overall immune state of the elderly. Secondly, the effectiveness and safety of regulating CD4^+^T cells through cytokines and growth factors, as well as CAR‐T therapy, still need to be further confirmed and corrected. Thirdly, in terms of drug therapy, the regulation of CD4^+^T cells by traditional Chinese medicine decoction and the active ingredients is a safe and promising direction that can be combined with emerging technologies such as nanotechnology in the future to improve the therapeutic effect. Fourthly, no matter what kind of treatment, the regulation of CD4^+^T cells to play an antiaging role and that can be achieved by regulating different phenotypes such as CD4^+^TN, CD4^+^Teff, CD4^+^Tm/TEMRA, etc., to ensure the health, longevity, and high immunity state of the elderly population. Removal of senescent cells can delay or alleviate many age‐related diseases. Senescent immune cells are the most dangerous senescent cell type, which will accelerate the aging process of other organs and the whole body. As the executive system responsible for immune surveillance and defense, the immune system plays a crucial role in governing the spatial and temporal distribution of immune cells. However, the existing regulation of immune function focuses on the analysis of immune cell subtype and number and lacks the technical system to elaborate its functional characteristics from the space–time dimension. Therefore, there is still much work to do to reveal the internal mechanisms involved in the regulation of immunosenescence.

## AUTHOR CONTRIBUTIONS

The review was designed by T.T.X., X.Z., and X.M.G.; literature collection and structural development were conducted by T.T.X. and Y.Z.; manuscript writing, and graphic/grammar checking were performed by T.T.X., J.Y.A., and Z.C.; manuscript revision was carried out by X.Q.Z., T.Y.C., and B.L.; X.Z. and X.M.G. actively participated in the supervising and editing of the manuscript. All authors have thoroughly reviewed and approved the final version of the manuscript.

## FUNDING INFORMATION

This work was supported by the National Key R&D Program of China [grant numbers 2022YFC3500300, 2022YFC3500305], Tianjin Municipal Education Commission Research Project [grant number 2022ZD039], and High‐level Discipline Construction Project of Traditional Chinese Medicine (Pharmacy), National Administration of Traditional Chinese Medicine.

## CONFLICT OF INTEREST STATEMENT

The authors declare no conflict of interest.

## Data Availability

Not applicable.
